# Diabetes, Disability, and Dementia Risk: Results From the HEPESE

**DOI:** 10.1093/geroni/igaa057.1906

**Published:** 2020-12-16

**Authors:** Elizabeth Vasquez, Meghana Gadgil, Weihu Zhang, Jacqueline L Angel

**Affiliations:** 1 University at Albany (SUNY), Rensselaer, New York, United States; 2 Dell Medical School - University of Texas, Austin, Texas, United States; 3 Albany University, Rensselaer, New York, United States; 4 The University of Texas at Austin, LBJ School of Public Affairs, austin, Texas, United States

## Abstract

The relationship between cognitive function, diabetes and disability among the oldest-old remains largely unexplored, particularly in the Latino population. This study examines dementia risk and diabetes status in a Mexican-origin older adult sample. The data are drawn from eight waves (1993 -2013) of the Hispanic Established Populations for the Epidemiologic Study of the Elderly (HEPESE; N=3,039, mean age at baseline=73.6 (±6.8)). We use multivariable Cox proportional hazards models to predict the relation between diabetes and time to incident dementia (MMSE<24, 1+ IADL), with risk adjustment for age of migration, socioeconomic status, acculturation, and health. Diabetes prevalence at baseline was 27.8 %. Diabetes was associated with a higher risk of developing dementia (HR)=1.22, p<0.001). Foreign-born older adults who migrated at ages 20- 49 had a higher survival probability of being dementia-free (HR=0.84, p=0.001). Our results further highlight the importance of evaluating differences in the cognitive outcomes of Mexican origin older adults.

